# Research involving participants and Brazilian General Data Protection Law

**DOI:** 10.1590/0034-7167-2025-0361

**Published:** 2026-07-27

**Authors:** Beatriz Bartulic Machado, Anna Luiza Bertin, Fernanda Miranda da Cruz, Thiago Ferreira de Araujo, Paula Midori Castelo

**Affiliations:** I Universidade Federal de São Paulo. São Paulo, São Paulo, Brazil; II Universidade Federal de São Paulo, Comitê de Ética em Pesquisa. São Paulo, São Paulo, Brazil

**Keywords:** Data Analysis, Privacy, Civil Rights, Data Management, Ethics, Research., Análisis de Datos, Privacidad, Derechos Civiles, Manejo de Datos, Ética en Investigación.

## Abstract

**Objectives:**

the constitution and use of personal databases for various purposes has increased in recent years, amplified by technological advancements. Recent regulations seek to keep pace with these advances and ensure the protection of individual’s privacy and dignity. The objective was to assess the impact of the Brazilian General Data Protection Law (LGPD) and Resolution 738/2024 of the Brazilian National Health Council on research with participants in Brazil.

**Methods:**

documentary research, using analysis of national and international regulations, laws and guidelines to describe the preand post-LGPD regulatory framework.

**Results:**

the law and Resolution 738/2024 represent important milestones for data protection and specific requirements for its use in research, in addition to reinforcing ethical observance and the need of informed consent.

**Final Considerations:**

these changes represent operational challenges, as well as dilemmas in balancing the importance of data use in health, education, and science, and the limits of individual protection.

## RESUMEN

**Objetivos:**

la creación y uso de bases de datos se han multiplicado en los últimos años. Las regulaciones buscan mantenerse al día con estos avances y garantizar la protección de la privacidad y dignidad de ciudadanos. El objetivo fue evaluar el impacto de la Ley General de Protección de Datos (LGPD) y Resolución 738/2024 del Consejo Nacional de Salud en la investigación con seres humanos en Brasil.

**Métodos:**

investigación documental de regulaciones, leyes y directrices nacionales e internacionales previo y posterior a LGPD.

**Resultados:**

la LGPD y Resolución 738/2024 representan hitos importantes para protección de datos, al imponer directrices específicas para su uso en investigación, además de reforzar la observancia ética y obtención del consentimiento libre e informado.

**Consideraciones Finales:**

Estos cambios representan desafíos y dilemas a la hora de equilibrar la importancia del uso de datos en salud, educación y ciencia con los límites de la protección individual.

## INTRODUCTION

Several data breach incidents and digital security violations have occurred in recent years, exposing sensitive personal data and raising concerns about how organizations store and protect personal information. Technology allows for collecting and processing data so efficiently and automatically in a way that traditional privacy protection approaches can be considered inadequate or ineffective. Therefore, special attention must be paid to the risk of abusive and segregative use of certain information, which privileges other information and reproduces patterns of prejudice and discrimination based on gender, race, and income, among others^(1)^.

Data, specifically personal data, has become a valuable asset, and the rapid technological advancement in data collection, processing, and sharing does not always keep pace with the development and publication of legal and ethical guidelines for its use. An example is the increasing digitization of documents and computerization, which leads to the need for new data security practices, such as transparency, accountability, management, and privacy. In response to the development of technologies and research designs, new policies and regulations for research involving human subjects have been implemented, making the entire process challenging for research institutions in Brazil and worldwide^(2)^.

In the field of clinical research with human subjects-development of new drugs, and the safety and efficacy of new treatments, medications, interventions, procedures, and devices-, researchers, sponsors, and managers handle sensitive data from research participants, such as sociodemographic data, health outcomes, and information on monitoring treatment safety and efficacy, from the moment of on-site collection, through data storage, processing, and sharing, culminating in the dissemination of results^(3)^.

The Brazilian General Data Protection Law (In Portuguese, *Lei Geral de Proteção de Dados Pessoais* - LGPD), Law 13,709^(4)^, came into force in September 2020 amid the COVID-19 pandemic, when the sharing and use of personal data between public and private entities intensified for the purposes of monitoring and containing the health crisis^(5,6)^. Gradually, it was implemented in sectors that directly or indirectly deal with personal and sensitive data, including health, assistance and research sectors^(7)^.

In February 2024, the Brazilian National Research Ethics Committee (In Portuguese, *Comissão Nacional de Pesquisa com Seres Humanos* - CONEP), subordinate to the Brazilian National Health Council (In Portuguese, *Conselho Nacional de Saúde* - CNS), published on its website a draft of Resolution 738/2024^(8)^, which came into force on January 21^st^, 2025. This resolution covers the use and constitution of databases for the purpose of scientific research involving human beings. The publication this resolution was long awaited by all professionals working in research with human beings, especially members of the Research Ethics Committees (part of the CEP/CONEP system).

## OBJECTIVES

To conduct a comprehensive analysis of the LGPD and CNS/CONEP Resolution 738/2024 Given the recent changes in the use and/or constitution of databases in research with human subjects and the dilemma between protecting individual privacy and producing knowledge for the advancement of health. The analysis of these regulatory texts highlights the impact of these two instruments on research with participants in Brazil in the coming years. Secondarily, the study contextualized the individual data protection within the scope of research with human subjects in Brazil before the LGPD.

## METHODS

This is a document analysis based on the READ approach^(9)^, which systematizes procedures for document analysis in health policy research. The research procedures are structured in four steps: (1) ready your materials, (2) extract data, (3) analyse data and (4) distil your findings.

In steps 1 and 2, data reading and extraction, the main documents selected to compose the *corpus* of the specific study were the LGPD and CNS/CONEP Resolution 738/2024. In addition to the two main documents, other national and international regulatory documents related to research with human beings and constitutional law were also selected, as illustrated in Chart 1, a non-exhaustive chart of regulatory documents.

**Chart 1:** Regulatory documents related to data protection in research with human participants included in this study and their respective sources (in chronological order)

Database	Source and website
Brazil, Ministry of Health	Nuremberg Code. 1947. Available at: https://bvsms.saude.gov.br/bvs/publicacoes/codigo_nuremberg.pdf ^(10)^
Federative Republic of Brazil	Federal Constitution of Brazil of 1988 [In Portuguese, *Constituição Federativa do Brasil de 1988 (Artigo 5º, inciso X)*]. Available at: https://www.planalto.gov.br/ccivil_03/constituicao/constituicao.htm ^(11)^
European Medicines Agency	ICH E 3: Structure and content of clinical study reports - Step 5, 1996. Available at: https://www.ema.europa.eu/en/ich-e3-structure-content-clinical-study-reports-scientific-guideline ^(3)^
Federative Republic of Brazil	Brazilian Civil Code, Law 10,406 of January 10, 2002 [In Portuguese, *Código Civil Brasileiro, Lei nº 10.406, de 10 de janeiro de 2002*]. Available at: http://www.planalto.gov.br/ccivil_03/leis/2002/L10406.htm ^(12)^
Food And Drug Administration (FDA)	Health Insurance Portability and Accountability Act. HIPAA Privacy Rule. Last version: 2003. Available at: https://www.hhs.gov/hipaa/for-professionals/privacy/laws-regulations/index.html ^(13)^
United Nations Educational, Scientific and Cultural Organization (UNESCO)	International Declaration on Human Genetic Data. UNESCO, 2004. Available at: https://bvsms.saude.gov.br/bvs/publicacoes/declaracao_inter_dados_genericos.pdf ^(14)^
United Nations Educational, Scientific and Cultural Organization (UNESCO)	Universal Declaration on Bioethics and Human Rights, 2006. Available at: https://unesdoc.unesco.org/ark:/48223/pf0000146180_por ^(15)^
Brazil, Ministry of Health	Decree 2,201 of September 14, 2011. Establishes the Brazilian National Guidelines for Biorepositories and Biobanks of Human Biological Material for Research Purposes [In Portuguese, *Portaria nº 2.201, de 14 de setembro de 2011. Estabelece as Diretrizes Nacionais para Biorrepositório e Biobanco de Material Biológico Humano com Finalidade de Pesquisa*]. Available at: https://bvsms.saude.gov.br/bvs/saudelegis/gm/2011/prt2201_14_09_2011.html ^(16)^
Brazilian National Research Ethics Committee (In Portuguese, *Comissão Nacional de Pesquisa com Seres Humanos* - CONEP)	Resolution 441 of May 12, 2011. Guidelines and regulatory standards for research involving human beings [In Portuguese, *Resolução nº 441, de 12 de maio de 2011. Diretrizes e normas regulamentadoras de pesquisas envolvendo seres humanos*]. Available at: https://bvsms.saude.gov.br/bvs/saudelegis/cns/2011/res0441_12_05_2011.html ^(17)^
Brazilian National Research Ethics Committee (In Portuguese, *Comissão Nacional de Pesquisa com Seres Humanos* - CONEP)	Circular Letter 039, dated July 19, 2011 [In Portuguese, *Carta Circular nº 039, de 19 de julho de 2011*]. Available at: https://www.gov.br/conselho-nacional-de-saude/pt-br/camaras-tecnicas-e-comissoes/conep/legislacao/cartas-circulares/carta-circular-no-039-de-19-de-julho-de-2011.pdf/view ^(18)^
Brazilian National Research Ethics Committee (In Portuguese, *Comissão Nacional de Pesquisa com Seres Humanos* - CONEP)	Resolution 466 of December 12, 2012. Guidelines and regulatory standards for research involving human beings. Brazilian National Health Council ((In Portuguese, *Conselho Nacional de Saúde* - CNS), Ministry of Health [In Portuguese, *Resolução nº 466, de 12 de dezembro de 2012. Diretrizes e normas regulamentadoras de pesquisas envolvendo seres humanos. Conselho Nacional de Saúde (CNS), Ministério da Saúde*]. Available at: https://www.gov.br/conselho-nacional-de-saude/pt-br/atos-normativos/resolucoes/2012/resolucao-no-466.pdf/view ^(19)^
Brazilian National Research Ethics Committee (In Portuguese, *Comissão Nacional de Pesquisa com Seres Humanos* - CONEP)	Resolution 510 of April 7, 2016. Resolution establishing the rules applicable to research in the Humanities and Social Sciences [In Portuguese, *Resolução nº 510, de 7 de abril de 2016. Resolução que dispõe sobre as normas aplicáveis a pesquisas em Ciências Humanas e Sociais*]. Available at: https://bvsms.saude.gov.br/bvs/saudelegis/cns/2016/res0510_07_04_2016.html ^(20)^
The International Council for Harmonisation of Technical Requirements for Pharmaceuticals for Human Use (ICH)	E6(R2) Good Clinical Practice: Integrated Addendum to ICH E6(R1). Available at: https://database.ich.org/sites/default/files/E6_R2_Addendum.pdf ^(21)^
European Union	General Data Protection Regulation (GDPR) - EU 2016/679. Available at: https://eur-lex.europa.eu/eli/reg/2016/679/oj/eng ^(22)^
Federative Republic of Brazil	Law 13,709 of August 14, 2018. Provides for the protection of personal data and amends Law 12,965 of April 23, 2014 (Brazilian Internet Bill of Rights) [In Portuguese, *Lei nº 13.709, de 14 de agosto de 2018. Dispõe sobre a proteção de dados pessoais e altera a Lei nº 12.965, de 23 de abril de 2014 (Marco Civil da Internet)*]. Available at: http://www.planalto.gov.br/ccivil_03/_ato2015-2018/2018/lei/L13709.htm ^(4)^
Brazilian Federal Council of Medicine (In Portuguese, *Conselho Federal de Medicina* - CFM)	CFM Resolution 2,217 of September 27, 2018. Code of Medical Ethics [In Portuguese, *Resolução CFM nº 2.217, de 27 de setembro de 2018. Código de Ética Médica*]. Available at: https://portal.cfm.org.br/images/PDF/cem2019.pdf ^(23)^
Brazilian National Data Protection Authority (In Portuguese, *Autoridade Nacional de Proteção de Dados* - ANPD)	Technical Study on the legal bases for personal data processing. Brasília, DF [In Portuguese, *Estudo Técnico sobre bases legais para o tratamento de dados pessoais. Brasília, DF*]. Available at: https://www.gov.br/anpd/pt-br/centrais-de-conteudo/documentos-tecnicos-orientativos/estudo-tecnico-a-lgpd-e-o-tratamento-de-dados-pessoais-para-fins-academicos-e-para-a-realizacao-de-estudos-por-orgao-de-pesquisa.pdf ^(24)^
Brazilian National Data Protection Authority (In Portuguese, *Autoridade Nacional de Proteção de Dados* - ANPD)	Guide ‘Processing of personal data for academic purposes and for conducting studies and research’, by Vargas *et al*., 2023. Brasília, DF [In Portuguese, *Guia orientativo ‘Tratamento de dados pessoais para fins acadêmicos e para a realização de estudos e pesquisas’, de Vargas et al., 2023*]. Brasília, DF. Available at: https://www.gov.br/anpd/pt-br/centrais-de-conteudo/materiais-educativos-e-publicacoes/web-guia-anpd-tratamento-de-dados-para-fins-academicos.pdf ^(25)^
Brazilian National Research Ethics Committee (In Portuguese, *Comissão Nacional de Pesquisa com Seres Humanos* - CONEP)	Resolution 738 of February 2024. Provides for the use of databases for scientific research involving human participants [In Portuguese, *Resolução nº 738, de Fevereiro de 2024. Dispõe sobre uso de bancos de dados com finalidade de pesquisa científica envolvendo seres humanos*]. Available at: https://www.gov.br/conselho-nacional-de-saude/pt-br/atos-normativos/resolucoes/2024/resolucao-no-738.pdf/view ^(8)^
World Medical Association (WMA)	WMA Declaration of Helsinki - Ethical Principles for Medical Research Involving Human Participants amended by the 75^th^ WMA General Assembly, Helsinki, Finland, October 2024. Available at: https://www.wma.net/policies-post/wma-declaration-of-helsinki/ ^(26)^

In step 3, data analysis, the documents were analyzed in light of previous research on “data protection” in the context of research with human subjects. For the bibliographic research, which underpins the analysis of the two central regulatory documents, structured searches were conducted in the PubMed and Google Scholar databases using the keywords “General Data Protection Law”, “Data Protection in Clinical Research”, and “CNS/CONEP Resolution 738/2024”. The inclusion criteria to support the proposed analysis and discussion were scientific articles, abstracts, book chapters, studies, and documents published in the last ten years related to the topic of data protection in research with human subjects.

Finally, in step 4, ethical and regulatory issues related to personal data protection in research were identified and discussed.

## RESULTS

Chart 1 provides a non-exhaustive list of national and international regulations, guidelines, codes, and legislation related to the topic studied and explored in depth in this study.

## DISCUSSION

Scientific articles and publications that discussed ethical and regulatory issues related to personal data protection in research, both before and after the LGPD, were included and discussed. This review provided a critical analysis of the regulatory framework existing before the LGPD and the construction of a narrative regarding individuals’ right to the privacy of their data, including in the clinical research conducted in Brazil.

The use of personal data for commercial purposes has driven the development of the legal framework for data protection. Currently, more than 128 countries have adopted some form of regulation or legislation to guarantee personal data privacy and protection^(27)^.

In the research involving human participants, data management is part of the everyday practice. The essence of research is the collection, processing, and analysis of sensitive health data and its sharing for the scientific assessment of the safety, efficacy, and quality of a health product or treatment so that it can be commercialized^(28)^. Personal data can include sensitive health, genetic, and biometric information, having invaluable worth for knowledge production. However, rigorous attention to protection to prevent risks to physical and mental integrity, health, honor, image, and privacy must be guaranteed^(20)^.

### National and international regulation of data use in research pre-Brazilian General Data Protection Law

The Nuremberg Code^(10)^, proposed in 1947, and the Declaration of Helsinki, whose first version was proposed in 1964^(29)^, guided ethical principles of research in signatory countries, emphasizing respect for autonomy, the voluntary consent of the human being as absolutely essential, taking precautions to protect the privacy of each research participant and the confidentiality of their personal data.

The Universal Declaration on Bioethics and Human Rights of the United Nations Educational, Scientific and Cultural Organization^(15)^ highlights that

The privacy of the persons concerned and the confidentiality of their personal information should be respected. To the greatest extent possible, such information should not be used or disclosed for purposes other than those for which it was collected or consented to, consistent with international law, in particular international human rights law (free translation).

With scientific advancements and the global dissemination of data and information, several countries have introduced regulations for data protection^(22,30)^. Recognizing different local laws and regulations is fundamental for international collaborations. Harmonization of data protection practices is necessary for conducting multicenter research, enabling the sharing of sensitive information without violating legal and ethical precepts^(22,30)^. Standardizing these practices should include Informed Consent Forms (ICFs), security measures, transparency and minimization of risk regarding the data collected, as well as the protection of the rights and privacy of participants.

In 1995, the Directive on the Protection of Personal Data of the European Parliament and of the Council (95/46/EC) aimed to standardize personal data collection, processing and use by the member states of the European Union. The regulatory document served as a reference for non-member countries and aimed to regulate and protect the free movement of personal data^(30)^. In 2016, Directive 95/46/EC was replaced by the General Data Protection Regulation (GDPR)^(22)^ to ensure a high level of individual protection and to eliminate obstacles to the free movement of personal data within the European Union. Implemented in 2018, the GDPR has become one of the most rigorous and comprehensive regulatory frameworks for data protection in the world, emphasizing the need for a balance between leveraging the value of data, protecting fundamental rights such as privacy and dignity, observing the requirement to obtain consent from participants and pseudonymization.

The United States Privacy Act of 1974^(31)^ was one of the main pieces of legislation to regulate the use of personal information by federal agencies. Although less restrictive than the European one, the American regulation raised the prospect that improper handling of personal data could cause significant harm to the individuals involved. Furthermore, it ensured the right of access to government databases, as well as the correction of information and accountability in case of data exposure^(30,31)^.

The U.S. Department of Health and Human Services published the Privacy Act to implement the Health Insurance Portability and Accountability Act of 1996 (HIPAA) requirements^(13)^. This law currently governs data protection in the country. HIPAA establishes standards for protecting health information and requirements to ensure data confidentiality, integrity, and availability. Despite its focus on health assistance, HIPAA guidelines also influence data protection practices in clinical research, promoting trust and integrity in information management and ensuring that sensitive information is protected against unauthorized access and use in clinical trials.

In Brazil, privacy protection was already foreseen in Article 5, item X, of the 1988 Federal Constitution^(11)^: “the intimacy, private life, honor and image of persons are inviolable, ensuring the right to compensation for material or moral damage resulting from their violation” (free translation). The Brazilian Civil Code also provides in Article 21 that “the private life of a natural person is inviolable, and the judge, at the request of the interested party, shall take the necessary measures to prevent or stop any act contrary to this rule” (free translation)^(12)^. In addition to these legal provisions, the CNS/CONEP Resolution 466/2012 also established guidelines for “using the material and data obtained in the research exclusively for the purpose foreseen in its protocol, or according to the participant’s consent” (free translation)^(19)^. These legal provisions, along with CNS/CONEP Resolution 466/2012, reinforced the importance of informed consent and personal data protection in conducting research with human beings even before the publication of the LGPD. The CNS/CONEP Resolution 466/2012 reiterates that “data obtained from research participants may not be used for purposes other than those stipulated in the protocol and/or in the informed consent” (free translation)^(19)^.

Concerning the use of patient record data, CONEP Circular Letter 039/2011^(18)^, which deals with research that uses data from patient records, highlights the importance of preserving data confidentiality, with emphasis on misuse. Patient records are documents that contain sensitive information, signs and images recorded by healthcare professionals relating to the physical-functional or mental state of a person^(32)^.

The data in the patient record is the sole and exclusive property of individuals, who provided this information under a confidential doctor-patient relationship for the purpose of their health treatment and care, and not for use in research (free translation).

The Brazilian Federal Council of Medicine (CFM Resolution 2,217 of September 2018) reinforces the protection of data contained in medical records and establishes that physicians are prohibited from allowing the handling and knowledge of medical records by persons not bound by professional secrecy (Article 85), as well as “releasing copies of the medical record in their custody, except to comply with a court order or for their own defense, as well as when written authorized by the patient” (free translation) (Article 89)^(23)^.

### Brazilian General Data Protection Law

The LGPD aims to regulate how personal data is used by individuals or companies to “protect the fundamental rights of freedom and privacy and the free development of the personality of the natural person” (free translation)^(4)^. The law, which came into effect in February 2020, aims to protect personal data, such as name, telephone number, home address, email, among others, as well as sensitive data, such as racial or ethnic origin, religious beliefs, public opinion, affiliation with religious, philosophical or political organizations, and health, sex life, genetic and biometric data^(33)^.

The LGPD was inspired by the GDPR with the aim to facilitating trade with the European Union and meeting Brazil’s interest in joining the Organisation for Economic Co-operation and Development, making legislative harmonization necessary^(30)^. In 2018, Provisional Measure 869/2018^(34)^ was enacted to regulate personal data protection in Brazil, creating the Brazilian National Data Protection Authority (In Portuguese, *Autoridade Nacional de Proteção de Dados* - ANPD) and defining rules for personal data processing in various situations, including academic research, the formulation of public policies or the provision of services by state bodies or their agents. In 2019, the ANPD was officially established as a direct public administration body, with the role, among others, of ensuring personal data protection under the LGPD, monitoring and applying sanctions in cases of data processing carried out in violation of legislation.

Compared to the 1988 Federal Constitution and the Brazilian Civil Code, the LGPD introduces new concepts that guarantee greater privacy, transparency, and security, highlighting citizens’ rights and giving them greater control over how their data will be used. The law also establishes guidelines, responsibilities, and obligations to be applied to any data processing operation carried out by a natural person or a legal entity under public or private law, regardless of the means, the country of its headquarters, or the country where the data is located.

Article 6 of the law establishes the essential principles for data processing. Firstly, purpose implies that personal data must be processed for legitimate, specific, explicit purposes, and participants must be informed. In the field of research, this means that the data collected must strictly serve the study objectives, as detailed in the protocol and the ICF. Another important point is limiting the use of data to what is strictly necessary to achieve the proposed objectives, i.e., collecting only relevant, proportionate, and non-excessive data. Furthermore, the free access, integrity, and transparency principles guarantee the right to easily access information about the form and duration of their data processing by the research participant. The law stipulates that security and risk prevention must also be guaranteed against unauthorized access, loss, alteration, communication, dissemination, or discriminatory, illicit, commercial, or abusive purposes that may harm individuals^(6,35)^.

Chapter II, “Personal data processing”, details the legal bases that legitimize personal data processing^(6)^. The law allows the processing of such data provided that anonymization is ensured and eliminates the need for explicit consent from participants if anonymization occurs in such a way that the results do not allow the identification of individuals:

In conducting public health studies, research institutions may have access to personal databases, which will be processed exclusively within the institution and strictly for the purpose of conducting studies and research, and maintained in a controlled and secure environment, in accordance with security practices provided for in specific regulations and which include, whenever possible, data anonymization or pseudonymization (free translation).

Thus, the data must be used exclusively for the specific purpose of the research and cannot be used for commercial or public purposes unrelated to the research project. Research institutions must implement robust technical and administrative measures to protect personal data throughout processing. The LGPD does not apply to anonymized data. However, if anonymization is reversed, the data becomes considered personal data and its use becomes regulated by law. Ramos *et al*.^(36)^ emphasize that re-identifying individuals when reversing data anonymization is illegal, although they note that complete anonymization of individual data is practically impossible.

### CNS/CONEP Resolution 738/2024

Resolution 738/2024 establishes specific guidelines aligned with the LGPD for the use and/or constitution of databases in research involving human beings^(8)^. The resolution respects the ethical principles already mentioned in CNS Resolution 466/2012, reaffirming the need to ensure the rights and confidentiality of participants and their information, as well as the researcher’s responsibility for the database and the security of this information. It also reiterates that research protocols involving the creation or use of databases must be processed through the CEP/CONEP system.

The resolution addresses the constitution and use of databases within the scope of research, processes that require detailed planning and consideration of multiple ethical and legal factors, especially ensuring that all data collected for research are obtained with due informed consent and from the provision of clear information about what the data is and how it will be used, stored and possibly shared with third parties in accordance with the purposes of the approved research^(8)^. Consent eliminates the consideration of misuse or exposure of individuals. However, as highlighted by Lima *et al*.^(32)^, to guarantee the honor, good name, or respectability of the person authorizing the use of consent, it is plausible to require that consent be free, clear, and specific for each purpose. Therefore, the broad, general, and unrestricted application of consent seems incorrect. Another aspect refers to the clear description of the security mechanisms to be implemented for accessing and storing data. As for the practice of accessing databases deposited in “data repositories” and sharing data between national and international research groups, according to Article 7, “personally identifying data must be removed, without exception, when data is stored, in whole or in part, in national or international databases, whether publicly or accessibly. Personally identifying data must be removed, without exception, when data is stored, in whole or in part, in national or international databases, whether publicly or accessibly” (free translation)^(8)^.

When the research protocol uses data from databases already established within the scope of the research, the researcher must present a statement of responsibility regarding the integrity, secrecy, and confidentiality of sensitive research participant data^(8)^. Possibilities for waiving the application of an ICF are foreseen with substantiated justification submitted to the CEP/CONEP system, such as in situations where the application of the ICF is justifiably unfeasible, when there is irreversible anonymization, i.e., data collected anonymously without identifying participants, or when there was a provision for the future and specified use of the data described in a previous ICF obtained in an approved project^(8)^.

For the use of databases created outside the scope of research (originating from hospitals, clinics, schools, among others), the resolution determines that, before any sharing for research purposes, the proposal must be reviewed by the CEP/CONEP system, since such data were not originally obtained for research^(18)^. For data not anonymized by the controller before sharing, obtaining participant consent through an ICF is mandatory^(8)^.

### Impact of the Brazilian General Data Protection Law and CNS/CONEP Resolution 738/2024 on research

The need to comply with the LGPD and the regulation of database use in research requires researchers, sponsors, Research Ethics Committees, and research and healthcare institutions to adapt to the new regulations. Thus, researchers must be qualified to act in accordance with data protection regulations. From the point of view of Research Ethics Committees, the entry into force of the LGPD brought challenges for its members and questions from the scientific community in the months following the publication of the law, which occurred in the midst of the COVID-19 pandemic, when the use of databases has intensified. The use of databases is a common practice in the scientific community that raises legal questions, especially when the requirement for the data subject’s consent is waived. Since the publication of the LGPD, educational, research, and care institutions have adapted and established procedures that ensure compliance with the legal requirements for data collection, storage, and use in research through the establishment of privacy offices and the appointment of legal data protection officers. The ANPD^(24)^ highlighted that

It can be stated that the LGPD sought to establish a balance between, on the one hand, the protection of personal data and guarantees of privacy and informational self-determination; and, on the other hand, academic freedom and the free flow of information necessary for conducting research in the most diverse areas of knowledge (free translation).

Obviously, managing and protecting large volumes of personal and sensitive data, as well as the need for data de-identification or pseudonymization, demands investments in infrastructure, security technologies, and specialized training that are fundamental to ensuring that participants’ information is secure and handled ethically. Furthermore, data processing can lead to the loss of information, thus limiting the use and usefulness of health information. This loss is sometimes necessary to protect the dignity and privacy of the individual and to conduct research responsibly. The balance between scientific advancement and the protection of individual rights requires that research protocols become more transparent and detailed.

### Study limitations

We can mention as limitations of this document analysis the small number of scientific publications on the subject, especially those that specifically address Brazilian legislation and the new regulations for the use of data in research with human beings, which is due to the recent nature of the law.

### Contributions to nursing, health, and public policy

This document analysis allows for a comprehensive assessment of the regulatory framework governing the handle and establishment of research databases involving human participants in Brazil, assisting professionals from all areas of health in the planning and development of projects, as well as in the conduct, execution, and security of research data involving human participants.

## FINAL CONSIDERATIONS

Brazilian regulations place Brazil on an international level of respect for sensitive data and privacy, similar to other jurisdictions, such as the GDPR in the European Union and HIPAA in the United States. The latest version of the Declaration of Helsinki^(37)^, published in October 2024, addresses, for the first time, the use of de-identified data in research in the following terms: “Physicians or other qualified individuals must obtain free and informed consent from research participants for the collection, processing, storage, and foreseeable secondary use of biological material and identifiable or re-identifiable data (...). A research ethics committee must approve the establishment and monitor ongoing use of such databases and biobanks”.

Finally, it is relevant to mention that the Law 14,874/2024^(38)^, published in the *Diário Oficial da União* on May 29, 2024, also known as the Clinical Research Law, came into effect on August 28, 2024. It establishes a legal framework for research with human beings in Brazil, although it is still in the process of being regulated through decrees and ordinances from the Ministry of Health. The chapter “On the Responsibilities of the Sponsor and the Researcher” mentions the need to “verify that the research participant has authorized direct access to their data and information for the purposes of monitoring, auditing, review by the competent ethics bodies and inspection by regulatory agencies” (free translation). Furthermore, Article 39 provides that

consent for the disposal of human biological material and data, during life or post-mortem, must be formalized through an Informed Consent Form and occur free of charge, altruistically, and with full information, respecting the provisions of Law 13,709 of August 14, 2018 (Brazilian General Data Protection Law) (free translation)

with particular emphasis on compliance with the LGPD. Furthermore, it stipulates a five-year period after the completion or discontinuation of the research for data storage by the institution executing the research and under the responsibility of the researcher, and emphasizes, in article 51, that “The institution executing the research must establish mechanisms to protect the confidentiality of information linked to the data, sharing only anonymous or coded data” (free translation).

## Data Availability

The research data are not available.
