# Supplementary material for: Research involving participants and Brazilian General Data Protection Law
Source: Rev Bras Enferm. 2026 Jul 27;79:e20250361. doi: 10.1590/0034-7167-2025-0361 (PMC13411320; doi:10.1590/0034-7167-2025-0361)
Supplement: Translation

# Translation: Pesquisa com seres humanos e Lei Geral de Proteção de Dados

**DOI:** 10.1590/0034-7167-2025-0361pt

**Authors:** Beatriz Bartulic Machado, Anna Luiza Bertin, Fernanda Miranda da Cruz, Thiago Ferreira de Araujo, Paula Midori Castelo

**Affiliations:** I Universidade Federal de São Paulo. São Paulo, São Paulo, Brasil; II Universidade Federal de São Paulo, Comitê de Ética em Pesquisa. São Paulo, São Paulo, Brasil

**Keywords:** Análise de Dados, Privacidade, Direitos Civis, Gerenciamento de Dados, Ética em Pesquisa.

## Abstract

**Objetivos:**

a constituição e uso de bancos de dados para fins variados tornaram-se massivos nos últimos anos, amparados pelo avanço tecnológico. Regulamentações recentes buscam acompanhar tais avanços e garantir a privacidade e dignidade dos cidadãos. Objetivou-se avaliar o impacto da Lei Geral de Proteção de Dados (LGPD) e da Resolução do Conselho Nacional de Saúde 738/2024 na pesquisa com seres humanos no Brasil.

**Métodos:**

pesquisa documental, utilizando análise de normativas, leis e diretrizes nacionais e internacionais para descrever o arcabouço regulatório prée pós-LGPD.

**Resultados:**

a LGPD e Resolução 738/2024 representam marcos importantes para a proteção de dados, impondo diretrizes específicas para uso na pesquisa, além de reforçarem a observância ética e obtenção do consentimento livre e esclarecido dos participantes.

**Considerações Finais:**

estas mudanças representam desafios operacionais e dilemas para equacionar a importância do uso de dados nas áreas da saúde, educação e ciência, e os limites da proteção individual.

## INTRODUÇÃO

Diversos incidentes de vazamento de dados e violações da segurança digital ocorreram nos últimos anos, expondo dados pessoais sensíveis e levantando preocupações sobre como as organizações armazenam e protegem informações pessoais. A tecnologia permite a coleta e processamento de dados de maneira tão eficaz e automática que as abordagens tradicionais de proteção da privacidade se mostram inadequadas ou ineficazes. Por isso, é preciso prestar atenção quanto ao risco de uso abusivo e segregatório de determinadas informações, que pode privilegiar pessoas ou grupos mas também reproduzir padrões de preconceito e discriminação de gênero, de raça e de renda, entre outros^(1)^.

Os dados, especificamente os dados pessoais, tornaram-se ativo valioso, e a rapidez do avanço tecnológico de coleta, processamento e compartilhamento de dados nem sempre acompanha o tempo de elaboração e publicação de diretrizes legais e éticas para seu uso. Exemplo disso é a crescente digitalização de documentos e informatização, que leva à necessidade de aplicação de práticas de segurança de dados que não existiam, como a transparência, responsabilidade, gerência e privacidade. Em resposta ao desenvolvimento de tecnologias e delineamentos de pesquisa, foram implementadas novas políticas e regulamentações para a pesquisa com seres humanos, o que torna todo o processo desafiador em instituições de pesquisa no Brasil e no mundo^(2)^.

No campo da pesquisa clínica com seres humanos - desenvolvimento de novos fármacos, avaliação da segurança e eficácia de novos tratamentos, medicamentos, intervenções, procedimentos e dispositivos - os pesquisadores, patrocinadores e gestores lidam com dados sensíveis de participantes de pesquisa, como dados sociodemográficos, desfechos de saúde e informações sobre o monitoramento de segurança e eficácia de tratamentos, desde o momento da coleta *in loco*, passando pelo armazenamento, processamento e compartilhamento de dados, que culminam na divulgação dos resultados^(3)^.

A Lei Geral de Proteção de Dados Pessoais (LGPD), Lei n^o^ 13.709^(4)^, entrou em vigor em setembro de 2020 em meio à pandemia de COVID-19, quando intensificaram-se o compartilhamento e a utilização de dados pessoais entre entidades públicas e privadas para fins de monitoramento e contenção da crise sanitária^(5,6)^. Gradativamente, foi implementada nos setores que, direta ou indiretamente, lidam com dados pessoais e sensíveis, incluindo setores de saúde, assistência e pesquisa^(7)^.

Em fevereiro de 2024, a Comissão Nacional de Pesquisa com Seres Humanos (CONEP), vinculada ao Conselho Nacional de Saúde (CNS), publicou em seu *website* uma minuta da Resolução CNS/CONEP nº 738/2024^(8)^, a qual entrou em vigor em 21/01/2025. Essa resolução dispõe sobre o uso e constituição de bancos de dados com finalidade de pesquisa científica envolvendo seres humanos. A publicação das diretrizes foi muito aguardada por todos os profissionais que atuam em pesquisa com seres humanos, em especial membros dos Comitês de Ética em Pesquisa integrantes do sistema CEP/CONEP.

## OBJETIVOS

Realizar uma análise abrangente da LGPD e da Resolução CNS/CONEP n^o^ 738/2024 diante das mudanças recentes no uso e/ou constituição de bancos de dados na pesquisa com seres humanos e do dilema entre a proteção da privacidade do indivíduo e a produção de conhecimento para o avanço da saúde. Na análise dos textos regulatórios, os impactos desses dois instrumentos para a pesquisa com seres humanos no Brasil nos próximos anos são destacados. Secundariamente, foi objeto de estudo contextualizar a proteção de dados individuais no âmbito da pesquisa com seres humanos no Brasil antes da LGPD.

## MÉTODOS

Esta é uma análise documental baseada na abordagem *READ approach* ^(9)^, que sistematiza procedimentos para uma análise de documentos na pesquisa em políticas de saúde. Os procedimentos de pesquisa estão estruturados em quatro etapas: 1) a leitura do material (*ready your materials*); 2) a extração de dados (*extract data*); 3) análise dos dados (*analyse data*); e 4) a discussão dos achados (*distil your findings*).

Nas etapas 1 e 2, leitura e extração dos dados, os documentos centrais selecionados para compor o *corpus* do estudo específico foram os textos da LGPD e da Resolução CNS/CONEP n°. 738/2024. Além destes dois documentos centrais, foram selecionados outros documentos regulatórios relativos à pesquisa com seres humanos e ao direito constitucional nacionais e internacionais, conforme ilustra o Quadro 1, um quadro não exaustivo de documentos regulatórios.

**Quadro 1:** Documentos regulatórios relacionados à temática de proteção de dados na pesquisa com seres humanos incluídos neste estudo e respectiva fonte para consulta (em ordem cronológica)

Base de dados	Fonte e endereço eletrônico
Brasil, Ministério da Saúde	Código de Nuremberg. 1947. Disponível em: https://bvsms.saude.gov.br/bvs/publicacoes/codigo_nuremberg.pdf ^(10)^
República Federativa do Brasil	Constituição Federativa do Brasil de 1988 (artigo 5º, inciso X). Disponível em: https://www.planalto.gov.br/ccivil_03/constituicao/constituicao.htm ^(11)^
*European Medicines Agency*	ICH E 3: *Structure and content of clinical study reports - Step 5*, 1996. Disponível em: https://www.ema.europa.eu/en/ich-e3-structure-content-clinical-study-reports-scientific-guideline ^(3)^
República Federativa do Brasil	Código Civil Brasileiro, Lei nº 10.406, de 10 de janeiro de 2002. Disponível em: http://www.planalto.gov.br/ccivil_03/leis/2002/L10406.htm ^(12)^
*Food And Drug Administration* (FDA)	*Health Insurance Portability and Accountability Act*. HIPAA *Privacy Rule*. Última revisão: 2003. Disponível em: https://www.hhs.gov/hipaa/for-professionals/privacy/laws-regulations/index.html ^(13)^
Organização das Nações Unidas para a Educação, a Ciência e a Cultura (UNESCO)	Declaração Internacional sobre os Dados Genéticos Humanos. UNESCO, 2004. Disponível em: https://bvsms.saude.gov.br/bvs/publicacoes/declaracao_inter_dados_genericos.pdf ^(14)^
Organização das Nações Unidas para a Educação, a Ciência e a Cultura (UNESCO)	Declaração Universal sobre Bioética e Direitos Humanos, 2006. Disponível em: https://unesdoc.unesco.org/ark:/48223/pf0000146180_por ^(15)^
Brasil, Ministério da Saúde	Portaria n.º 2.201, de 14 de setembro de 2011. Estabelece as Diretrizes Nacionais para Biorrepositório e Biobanco de Material Biológico Humano com Finalidade de Pesquisa https://bvsms.saude.gov.br/bvs/saudelegis/gm/2011/prt2201_14_09_2011.html ^(16)^
Comissão Nacional de Ética em Pesquisa (CONEP)	Resolução nº 441, de 12 de maio de 2011. Diretrizes e normas regulamentadoras de pesquisas envolvendo seres humanos. https://bvsms.saude.gov.br/bvs/saudelegis/cns/2011/res0441_12_05_2011.html ^(17)^
Comissão Nacional de Ética em Pesquisa (CONEP)	Carta Circular nº 039, de 19 de julho de 2011. Disponível em: https://www.gov.br/conselho-nacional-de-saude/pt-br/camaras-tecnicas-e-comissoes/conep/legislacao/cartas-circulares/carta-circular-no-039-de-19-de-julho-de-2011.pdf/view ^(18)^
Comissão Nacional de Ética em Pesquisa (CONEP)	Resolução nº 466, de 12 de dezembro de 2012. Diretrizes e normas regulamentadoras de pesquisas envolvendo seres humanos. Conselho Nacional de Saúde (CNS), Ministério da Saúde. Disponível em: https://www.gov.br/conselho-nacional-de-saude/pt-br/atos-normativos/resolucoes/2012/resolucao-no-466.pdf/view ^(19)^
Comissão Nacional de Ética em Pesquisa (CONEP)	Resolução nº 510, de 7 de abril de 2016. Resolução que dispõe sobre as normas aplicáveis a pesquisas em Ciências Humanas e Sociais. Disponível em: https://bvsms.saude.gov.br/bvs/saudelegis/cns/2016/res0510_07_04_2016.html ^(20)^
Conselho Internacional para Harmonização de Requisitos Técnicos para Produtos Farmacêuticos de Uso Humano (ICH)	E6(R2) *Good Clinical Practice: Consolidated Guideline 2016*. Adendo Integrado ao ICH E6 (R1): guia de boas práticas clínicas E6(R2). Disponível em: https://database.ich.org/sites/default/files/E6_R2_Addendum.pdf ^(21)^
União Europeia	*General Data Protection Regulation* (GDPR) - EU 2016/679. Disponível em https://eur-lex.europa.eu/eli/reg/2016/679/oj/eng ^(22)^
República Federativa do Brasil	Lei nº 13.709, de 14 de agosto de 2018. Dispõe sobre a proteção de dados pessoais e altera a Lei nº 12.965, de 23 de abril de 2014 (Marco Civil da Internet). Diário Oficial da União: seção 1, Brasília, DF, 15 ago. 2018. Disponível em: http://www.planalto.gov.br/ccivil_03/_ato2015-2018/2018/lei/L13709.htm ^(4)^
Conselho Federal de Medicina (CFM)	Resolução CFM nº 2.217, de 27 de setembro de 2018. Código de Ética Médica. Disponível em: https://portal.cfm.org.br/images/PDF/cem2019.pdf ^(23)^
Autoridade Nacional de Proteção de Dados (ANPD)	Estudo Técnico sobre bases legais para o tratamento de dados pessoais. Brasília, DF. Disponível em: https://www.gov.br/anpd/pt-br/centrais-de-conteudo/documentos-tecnicos-orientativos/estudo-tecnico-a-lgpd-e-o-tratamento-de-dados-pessoais-para-fins-academicos-e-para-a-realizacao-de-estudos-por-orgao-de-pesquisa.pdf ^(24)^
Autoridade Nacional de Proteção de Dados (ANPD)	Guia orientativo ‘Tratamento de dados pessoais para fins acadêmicos e para a realização de estudos e pesquisas’, de Vargas *et al*., 2023. Brasília, DF. Disponível em: https://www.gov.br/anpd/pt-br/centrais-de-conteudo/materiais-educativos-e-publicacoes/web-guia-anpd-tratamento-de-dados-para-fins-academicos.pdf ^(25)^
Comissão Nacional de Ética em Pesquisa (CONEP)	Resolução nº 738, de fevereiro de 2024. Dispõe sobre uso de bancos de dados com finalidade de pesquisa científica envolvendo seres humanos. Disponível em: https://www.gov.br/conselho-nacional-de-saude/pt-br/atos-normativos/resolucoes/2024/resolucao-no-738.pdf/view ^(8)^
*World Medical Association* (WMA)	*WMA Declaration of Helsinki - Ethical Principles for Medical Research Involving Human Participants amended by the 75^th^ WMA General Assembly, Helsinki, Finland, October 2024*. Disponível em: https://www.wma.net/policies-post/wma-declaration-of-helsinki/ ^(26)^

Na etapa 3, análise dos dados, os documentos foram analisados à luz de pesquisas prévias sobre o tema da “proteção de dados” no âmbito da pesquisa com seres humanos. Para a pesquisa bibliográfica, que fundamenta as análises dos dois documentos regulatórios centrais, realizaram-se buscas estruturadas nas bases de dados *PubMed* e *Google* Acadêmico, utilizando como palavras-chave “Lei Geral de Proteção de Dados”, “Proteção de Dados em Pesquisa Clínica” e “Resolução CNS/CONEP n^o^ 738/2024”. Os critérios de inclusão foram artigos científicos, resumos, capítulos de livros, estudos e documentos publicados nos últimos dez anos relativos à temática de proteção de dados na pesquisa com seres humanos.

Por fim, na etapa 4, foram identificadas e discutidas questões éticas e regulatórias relacionadas à proteção de dados pessoais em pesquisas.

## RESULTADOS

O Quadro 1 traz uma lista não exaustiva das regulamentações, diretrizes, códigos e legislações nacionais e internacionais relacionados ao tema estudado e aprofundados neste estudo.

## DISCUSSÃO

Artigos científicos e publicações que abordavam questões éticas e regulatórias relacionadas à proteção de dados pessoais em pesquisa, tanto no período pré-LGPD quanto no pós-LGPD, foram incluídos e discutidos. A análise documental realizada proporcionou uma análise crítica do repertório regulatório existente antes da LGPD e a construção de uma narrativa a respeito do direito da pessoa natural à privacidade de seus dados, incluindo a pesquisa clínico-científica realizada no Brasil.

O uso de dados pessoais para fins comerciais impulsionou o desenvolvimento do quadro jurídico em termos de proteção de dados. Atualmente, mais de 128 países já adotaram alguma normativa ou legislação para garantir a privacidade e proteção de dados pessoais^(27)^.

Na pesquisa com seres humanos, a manipulação de dados faz parte do cotidiano da prática. A essência da pesquisa é a coleta, processamento e análise de dados sensíveis de saúde, bem como seu compartilhamento, para a comprovação científica da segurança, eficácia e qualidade de um produto ou tratamento de saúde, para que este possa ser comercializado^(28)^. Os dados pessoais podem abranger informações sensíveis de saúde, genéticas e biométricas, tendo inestimável valor para a produção de conhecimento. No entanto, a atenção à proteção rigorosa para prevenir riscos à integridade física e psíquica, à saúde, à honra, à imagem e à privacidade deve ser garantida^(20)^.

### Regulamentação nacional e internacional do uso de dados em pesquisa pré-Lei Geral de Proteção de Dados Pessoais

O Código de Nuremberg^(10)^, proposto em 1947, e a Declaração de Helsinki, cuja primeira versão foi proposta em 1964^(29)^, nortearam princípios éticos da pesquisa nos países signatários, com ênfase ao respeito à autonomia, ao consentimento voluntário do ser humano como absolutamente essencial, à tomada de precauções para proteger a privacidade de cada sujeito de investigação e à confidencialidade dos seus dados pessoais.

A Declaração Universal sobre Bioética e Direitos Humanos da Organização da Organização das Nações Unidas para a Educação, a Ciência e a Cultura^(15)^ destaca que

A vida privada das pessoas em causa e a confidencialidade das informações que lhes dizem pessoalmente respeito devem ser respeitadas. Tanto quanto possível, tais informações não devem ser utilizadas ou difundidas para outros fins que não aqueles para que foram coligidos ou consentidos, e devem estar em conformidade com o direito internacional, e nomeadamente com o direito internacional relativo aos direitos humanos.

Com o avanço técnico-científico e a disseminação de dados e de informações em termos globais, diversos países introduziram regulamentações para a proteção de dados^(22,30)^. Reconhecer as diferentes legislações e normativas locais é fundamental para colaborações internacionais. A harmonização das práticas de proteção de dados se faz necessária para a realização de pesquisas multicêntricas, viabilizando o compartilhamento de informações sensíveis sem violar os preceitos legais e éticos^(22,30)^. O nivelamento dessas práticas deve incluir o Termo de Consentimento Livre e Esclarecido (TCLE), medidas de segurança, transparência e minimização de risco dos dados coletados, além da proteção dos direitos e da privacidade dos participantes.

Em 1995, a Diretiva de Proteção de Dados Pessoais do Parlamento Europeu e Conselho (95/46/CE) objetivou uniformizar a coleta, o tratamento e o uso de dados pessoais pelos estados membros da União Europeia. O documento regulatório serviu como referência para países não membros, e teve o intuito de regular e proteger a livre circulação dos dados pessoais^(30)^. Em 2016, a Diretiva 95/46/CE deu lugar à *General Data Protection Regulation* (GDPR)^(22)^ para assegurar um nível de proteção elevado às pessoas singulares e eliminar os obstáculos à circulação de dados pessoais na União Europeia. Implementado em 2018, o GDPR tornou-se um dos marcos regulatórios mais rigorosos e abrangentes para a proteção de dados no mundo, enfatizando a necessidade de equilíbrio entre o aproveitamento do valor dos dados, a proteção dos direitos fundamentais, como privacidade e dignidade, a observância da obtenção do consentimento dos participantes e a pseudoanonimização.

A Lei da Privacidade dos Estados Unidos de 1974^(31)^ foi uma das principais legislações a regulamentar o uso de informações pessoais por órgãos federais. Embora menos restritiva que a europeia, a regulamentação americana levantou a perspectiva de que o tratamento inadequado de dados pessoais poderia causar danos significativos aos indivíduos envolvidos. Além disso, assegurou o direito de acesso aos bancos de dados governamentais, bem como a correção de informações e responsabilização em caso de exposição dos dados^(30,31)^.

O Departamento de Saúde e Serviços Humanos dos Estados Unidos da América publicou a Lei da Privacidade para implementar os requisitos da Lei de Portabilidade de Seguro de Saúde de 1996 (em inglês, *Health Insurance Portability and Accountability Act of 1996*, HIPAA)^(13)^. Tal lei atualmente rege a proteção de dados no país. A HIPAA estabelece padrões para proteção de informações de saúde e requisitos para garantir a confidencialidade, integridade e disponibilidade dos dados. Apesar do foco na assistência à saúde, as diretrizes da HIPAA também exercem influência nas práticas de proteção de dados em pesquisa clínica, promovendo a confiança e a integridade no gerenciamento de informações e garantindo que informações sensíveis sejam protegidas contra o acesso e o uso não autorizados em ensaios clínicos.

No Brasil, a proteção da privacidade já estava prevista no artigo 5º, inciso X, da Constituição Federal de 1988^(11)^: “são invioláveis a intimidade, a vida privada, a honra e a imagem das pessoas, assegurado o direito à indenização pelo dano material ou moral decorrente de sua violação”. O Código Civil também prevê no artigo 21° que “a vida privada da pessoa natural é inviolável, e o juiz, a requerimento do interessado, adotará as providências necessárias para impedir ou fazer cessar ato contrário a esta norma”^(12)^. Além desses dispositivos legais, as diretrizes da Resolução CNS/CONEP nº 466/2012 também estabeleciam orientações para “utilizar o material e os dados obtidos na pesquisa exclusivamente para a finalidade prevista no seu protocolo, ou conforme o consentimento do participante”^(19)^. Tais dispositivos legais, juntamente com a Resolução CNS nº 466/2012, reforçavam a importância do consentimento informado e da proteção dos dados pessoais na condução de pesquisas com seres humanos antes mesmo da publicação da LGPD. A Resolução nº 466/2012 reitera que “os dados obtidos a partir dos participantes da pesquisa não poderão ser usados para outros fins além dos previstos no protocolo e/ou no consentimento livre e esclarecido”^(19)^.

Com relação ao uso de dados de prontuários de pacientes, a Carta Circular CONEP nº 039/2011^(18)^, que trata de pesquisas que se utilizam de dados provenientes de prontuários de paciente, destaca a importância de preservar a confidencialidade dos dados, com destaque para o desvio de finalidade.

Os prontuários são documentos pessoais que contêm informações, sinais e imagens registrados pelos profissionais de saúde relativos ao estado físico-funcional ou mental de uma pessoa^(32)^:

Os dados do prontuário são de propriedade única e exclusiva do próprio sujeito, que forneceu tais informações em uma relação de confidencialidade entre médico e paciente, para realização do seu tratamento e cuidado médicos, e não para utilização de tais dados em pesquisas.

O Código de Ética Médica (Resolução CFM nº, de 2.217 de setembro de 2018) reforça a proteção dos dados contidos em prontuários e estabelece que é vedado ao médico permitir o manuseio e o conhecimento dos prontuários por pessoas não obrigadas ao sigilo profissional (artigo 85^o^), bem como “liberar cópias do prontuário sob sua guarda, exceto para atender a ordem judicial ou para sua própria defesa, assim como quando autorizado por escrito pelo paciente” (artigo 89^o^)^(23)^.

### A Lei Geral de Proteção de Dados Pessoais

A LGPD visa regulamentar a maneira pela qual os dados pessoais são utilizados por pessoas ou empresas, para “proteger os direitos fundamentais de liberdade e de privacidade e o livre desenvolvimento da personalidade da pessoa natural”^(4)^. A lei, que entrou em vigor em fevereiro de 2020, tem como objetivo proteger dados pessoais, como nome, contato telefônico, endereço residencial, correio eletrônico, entre outros, além de dados sensíveis, como origem racial ou ética, convicção religiosa, opinião pública, filiação a organizações de caráter religioso, filosófico ou político, e dados de saúde, de vida sexual, genéticos e biométricos^(33)^.

A LGPD foi inspirada na legislação europeia (GDPR) com vistas a facilitar as trocas comerciais com a União Europeia e atender ao interesse do Brasil em integrar a Organização para Cooperação e Desenvolvimento Econômico, tornando necessária a harmonização legislativa^(30)^. Em 2018, foi promulgada a Medida Provisória n^o^ 869/2018^(34)^ para regulamentar a proteção de dados pessoais no Brasil, com a criação da Autoridade Nacional de Proteção de Dados (ANPD) e a definição de regras para o tratamento de dados pessoais em diversas situações, incluindo a pesquisa acadêmica, a formulação de políticas públicas ou a prestação de serviços por órgãos estatais ou por seus prepostos. Em 2019, foi instaurada efetivamente a ANPD como órgão de administração pública direta, com o papel, entre outros, de zelar pela proteção dos dados pessoais nos termos da LGPD, fiscalizar e aplicar sanções em caso de tratamento de dados realizado em descumprimento à legislação.

Comparada à Constituição Federal de 1988 e o Código Civil Brasileiro, a LGPD apresenta novos conceitos que garantem maior privacidade, transparência e segurança, destacando os direitos dos cidadãos e dando a eles maior controle sobre como seus dados serão utilizados. A lei também estabelece diretrizes, responsabilidades e obrigações a serem aplicadas a qualquer operação de tratamento realizada por pessoa natural ou por pessoa jurídica de direito público ou privado, independentemente do meio, do país de sua sede ou do país onde estejam localizados os dados.

No artigo 6°, a lei estabelece os princípios essenciais para o tratamento de dados. Primeiramente, a finalidade implica que os dados pessoais devem ser tratados para propósitos legítimos, específicos, explícitos e informados ao titular. No campo da pesquisa, significa que os dados coletados devem servir estritamente aos objetivos do estudo, conforme detalhado no protocolo e no TCLE. Outro ponto importante é a limitação da utilização dos dados ao que é estritamente necessário para alcançar os objetivos propostos, ou seja, coletar apenas os dados pertinentes, proporcionais e não excessivos. Além disso, os princípios de livre acesso, integralidade e transparência garantem aos titulares o direito de acessar facilmente informações sobre a forma e a duração do tratamento de seus dados. A lei prevê que a segurança e a prevenção de riscos também devem ser garantidas contra acessos não autorizados, perda, alteração, comunicação, difusão ou fins discriminatórios, ilícitos, comerciais ou abusivos que possam prejudicar os participantes^(6,35)^.

O capítulo II, “Tratamento de dados pessoais”, detalha as bases legais que legitimam o tratamento de dados pessoais^(6)^. A lei permite o tratamento desses dados desde que seja assegurada sua anonimização, e elimina a necessidade de consentimento explícito dos titulares caso a anonimização ocorra de forma que os resultados não permitam a identificação dos indivíduos:

Na realização de estudos em saúde pública, os órgãos de pesquisa poderão ter acesso a bases de dados pessoais, que serão tratados exclusivamente dentro do órgão e estritamente para a finalidade de realização de estudos e pesquisas e mantidos em ambiente controlado e seguro, conforme práticas de segurança previstas em regulamento específico e que incluam, sempre que possível, a anonimização ou pseudoanonimização dos dados.

Assim, os dados devem ser utilizados exclusivamente para o propósito específico da pesquisa e não podem ser aproveitados para finalidades comerciais ou públicas não relacionadas ao projeto de pesquisa. Já os órgãos de pesquisa devem implementar medidas técnicas e administrativas robustas para proteger dados pessoais durante todo o processo de tratamento. A LGPD não se aplica a dados anonimizados. Entretanto, se a anonimização for revertida, os dados passam a ser considerados dados pessoais, e seu uso torna-se regulado pela lei. Ramos *et al*.^(36)^ destacam que a reidentificação das pessoas na reversão da anonimização de dados é uma contravenção, embora façam a ressalva de que hoje o anonimato completo dos dados individuais é praticamente impossível.

### A Resolução CNS/CONEP n^o^ 738/2024

A Resolução nº 738/2024 estabelece diretrizes específicas alinhadas à LGPD para uso e/ou constituição de bancos de dados em pesquisas envolvendo seres humanos^(8)^. A resolução respeita os princípios éticos já citados na Resolução CNS n^o^ 466/2012, reafirmando a necessidade de assegurar os direitos e a confidencialidade dos participantes e suas informações, assim como a responsabilidade do pesquisador com o banco de dados e com a segurança dessas informações. Reitera também que os protocolos de pesquisa que envolvem a constituição ou uso de bancos de dados devem tramitar pelo sistema CEP/CONEP.

A resolução aborda a constituição e o uso de bancos de dados no âmbito da pesquisa, processos que requerem um planejamento detalhado e a consideração de múltiplos fatores éticos e legais, em especial a garantia de que todos os dados coletados para a pesquisa sejam obtidos com o devido consentimento informado e a partir do fornecimento de informações claras sobre quais são os dados e como serão utilizados, armazenados e, possivelmente, compartilhados com terceiros, em conformidade com os propósitos da pesquisa aprovada^(8)^. O consentimento afasta a consideração do uso indevido ou de exposição do indivíduo. No entanto, como destacado por Lima *et al*.^(32)^, para garantir a honra, a boa fama ou a respeitabilidade daquele que autoriza, é plausível exigir que o consentimento seja livre, claro e específico para cada fim. Desse modo, parece incorreta a aplicação ampla, geral e irrestrita de consentimento. Outro aspecto refere-se à descrição clara dos mecanismos de segurança a serem implementados para acesso e armazenamento dos dados. Sobre a prática de acesso a bancos de dados depositados em “repositórios de dados” e compartilhamento de dados entre grupos de pesquisa nacionais e internacionais, segundo o artigo 7º, “os dados pessoais identificadores deverão ser removidos, obrigatoriamente, quando houver depósito dos dados, de forma parcial ou total, em bancos nacionais ou internacionais, de acesso público ou restrito”^(8)^.

Quando o protocolo de pesquisa utilizar dados provenientes de bancos já constituídos no âmbito da pesquisa, deve ser apresentado um termo de responsabilidade do pesquisador pela integridade, sigilo e confidencialidade no uso de dados sensíveis dos participantes da pesquisa^(8)^. Possibilidades de dispensa de aplicação do TCLE estão previstas com justificativa consubstanciada submetida ao sistema CEP/CONEP, como em situações em que a aplicação do TCLE é justificadamente inviável, quando há anonimização irreversível, ou seja, coletados anonimamente sem a identificação do participante, ou quando houve a previsão do uso futuro e especificado dos dados descrita em TCLE anterior obtido em projeto aprovado^(8)^.

Para uso de bancos de dados constituídos fora do âmbito da pesquisa (oriundos de hospitais, clínicas, escolas, entre outros), a resolução determina que, antes de qualquer compartilhamento para fins de pesquisa, é obrigatória a apreciação da proposta pelo sistema CEP/CONEP, uma vez que tais dados não foram obtidos originalmente para pesquisa^(18)^. Para dados não anonimizados pelo controlador antes do compartilhamento, é obrigatória a obtenção do consentimento do participante por meio do TCLE^(8)^.

### Impacto da Lei Geral de Proteção de Dados Pessoais e da Resolução CNS/CONEP n^o^ 738/2024 para a pesquisa

A necessidade de conformidade com a LGPD e com a regulamentação do uso de banco de dados em pesquisa exige que pesquisadores, patrocinadores, Comitês de Ética em Pesquisa e instituições de pesquisa e saúde se adequem às novas normativas. Assim, investigadores devem possuir qualificação para atuar em conformidade com as normativas de proteção de dados.

Do ponto de vista dos Comitês de Ética em Pesquisa, a entrada em vigor da LGPD trouxe desafios para seus membros e questionamentos da comunidade científica nos meses subsequentes à publicação da lei, que ocorreu em meio à pandemia de COVID-19, quando o uso de bancos de dados se intensificou. Prática comum no meio científico, o uso de bancos de dados suscita questões de ordem jurídica, principalmente quando envolve a dispensa do termo de consentimento do titular dos dados. Desde a publicação da LGPD, instituições de ensino, pesquisa e assistência têm se adaptado e instituído procedimentos que assegurem o cumprimento dos requisitos legais para coleta, armazenamento e uso de dados em pesquisa por meio da instauração de escritórios de privacidade e nomeação dos encarregados legais de proteção de dados. Conforme ressalta a ANPD^(24)^,

Pode-se afirmar que a LGPD procurou estabelecer uma relação de equilíbrio entre, de um lado, a proteção de dados pessoais e as garantias da privacidade e da autodeterminação informativa; e, de outro lado, a liberdade acadêmica e o livre fluxo de informações necessário para a realização de pesquisas nas mais diversas áreas do saber.

Obviamente, o gerenciamento e a proteção de grandes volumes de dados pessoais e sensíveis, além da necessidade de desidentificação ou pseudoanonimização de dados, demandam investimentos em infraestrutura, tecnologias de segurança e treinamento especializado fundamentais para garantir que as informações dos participantes estejam seguras e sejam tratadas de forma ética. Ainda, o tratamento dos dados pode levar à perda de informações, limitando, assim, o uso e a utilidade das informações de saúde. Esse prejuízo, por vezes, é necessário para proteger a dignidade e a privacidade do indivíduo e conduzir a pesquisa de forma responsável. O equilíbrio entre o avanço científico e a proteção de direitos individuais exige que os protocolos de pesquisa se tornem mais transparentes e detalhados.

### Limitações do estudo

Podemos citar como limitações desta análise documental o pequeno número de publicações científicas acerca do tema, sobretudo as que abordem especificamente a legislação brasileira e as novas normativas para o uso de dados em pesquisa com seres humanos, o que se deve ao caráter recente da lei.

### Contribuições para a área da enfermagem, saúde ou políticas públicas

Esta análise documental permite uma avaliação abrangente do arcabouço normativo que rege o uso e a constituição de bancos de dados de pesquisa com seres humanos no Brasil, auxiliando profissionais de todas as áreas da saúde no planejamento e elaboração de projetos de pesquisa, assim como na condução, execução e segurança de dados de pesquisa com seres humanos.

## CONSIDERAÇÕES FINAIS

A regulamentação brasileira coloca o Brasil em um patamar internacional de respeito aos dados sensíveis e à privacidade, similar ao de outras jurisdições, como a GDPR, na União Europeia, e a HIPAA, nos Estados Unidos. A última versão da Declaração de Helsinque^(37)^, publicada em outubro de 2024, aborda, pela primeira vez, o uso de dados desidentificados em pesquisa nos seguintes termos: “Os médicos ou outros indivíduos qualificados devem obter consentimento livre e informado dos participantes da pesquisa para a coleta, processamento, armazenamento e uso secundário previsível de material biológico e dados identificáveis ou reidentificáveis (...). Um Comitê de Ética em Pesquisa deve aprovar o estabelecimento e monitorar o uso contínuo de tais bancos de dados e biobancos” (tradução livre).

É relevante mencionar que a Lei n^o^ 14.874/2024^(38)^, publicada no Diário Oficial da União em 29 de maio de 2024, também conhecida como Lei de Pesquisa Clínica, passou a vigorar em 28 de agosto de 2024. Ela estabelece um marco legal para pesquisas com seres humanos no Brasil, embora ainda esteja em fase de regulamentação por meio de decretos e portarias do Ministério da Saúde. No capítulo “Das Responsabilidades do Patrocinador e do Pesquisador”, cita-se a necessidade de “verificação de que o participante da pesquisa tenha autorizado o acesso direto a seus dados e a suas informações para fins de monitoramento, de auditoria, de revisão pelas entidades éticas competentes e de inspeção de agências reguladoras”. Ainda, o artigo 39º dispõe que

o consentimento para a disposição de material biológico humano e de seus dados, em vida ou post mortem, deverá ser formalizado por meio de TCLE e ocorrer de forma gratuita, altruísta e esclarecida, respeitadas as disposições da Lei nº 13.709, de 14 de agosto de 2018 (Lei Geral de Proteção de Dados Pessoais),

com destaque à observância da LGPD. Ainda, estipula o prazo de cinco anos após o término ou a descontinuidade da pesquisa para armazenamento de dados pela instituição executora da pesquisa e sob responsabilidade do pesquisador e reitera, no artigo 51, que “A instituição executora da pesquisa deverá estabelecer mecanismos para proteger a confidencialidade das informações vinculadas aos dados, compartilhando apenas dados anônimos ou codificados”.

## Data Availability

Os dados de pesquisa não estão disponíveis.
